# OPTILATER: optimal long-term survival after cancer – a cross-sectional study protocol for a quantitative survey on the care situation of long-term cancer survivors in Germany

**DOI:** 10.1186/s12885-025-15096-7

**Published:** 2025-10-24

**Authors:** C. N. Martin, N. De Lazzari, J. Kersten, K. Claassen, C. Jansen, K. Kaminski, F. Baumann, M. Götte, S. Palm, A. Stang, V. Grünwald, U. Dirksen, M. A. Teufel, E.-M. Skoda

**Affiliations:** 1 Clinic for Psychosomatic Medicine und Psychotherapy, LVR-University Hospital Essen, Essen, North Rhine-Westphalia Germany; 2 Center for Translational Neuro-and Behavioral Sciences (C-TNBS), University of Duisburg-Essen, Essen, North Rhine-Westphalia Germany; 3 Department of Palliative Medicine, University Hospital Essen, West German Cancer Center Essen, Essen, North Rhine-Westphalia Germany; 4 University Hospital Essen, West German Cancer Center Essen, Registry, Essen, North Rhine-Westphalia Germany; 5 University Hospital Essen, West German Cancer Center Essen, Board of Directors, Essen, North Rhine-Westphalia Germany; 6 Department of Pediatrics III (Oncology, Hematology, Cardiology, Rheumatology, Pulmonology), University Hospital Essen, Essen, North Rhine- Westphalia Germany; 7 Department of Urology, University Hospital Essen, Essen, North Rhine- Westphalia Germany; 8 Institute of Medical Informatics, Biometry and Epidemiology, University Hospital Essen, Essen, North Rhine-Westphalia Germany; 9 Deparment I of Internal Medicine, Center of Integrated Oncology, University Hospital Cologne, Cologne, North Rhine-Westphalia Germany; 10 Cancer Registry of North-Rhine Westphalia, Bochum, North Rhine-Westphalia Germany; 11 Department of Human Medicine, Medical School Hamburg, Hamburg, Hamburg, Germany

**Keywords:** Cancer survivors, Long-term care, Health-related quality of life, Quantitative interviews, Survivorship care, Migration background, Cancer care barriers, Germany, Needs-based care, Somatic late effects

## Abstract

**Background:**

Cancer survivors in Germany face considerable challenges related to the late and long-term effects of treatment and a lack of post-treatment support. Despite an increasing number of cancer survivors, existing healthcare systems are insufficiently adapted to meet their ongoing needs, particularly for long-term survivors who may experience physical, emotional, and socio-economic hardships. This study aims to address the knowledge gaps in the care situation of long-term cancer survivors, focusing on their experiences and the barriers they face in accessing care.

**Methods:**

This study protocol outlines the methodology for a quantitative survey involving up to 3,300 long-term cancer survivors across various cancer types in Germany. The survey assesses their experiences with cancer care, focusing on diet, exercise, mental health, sleep, cognition, overall health-related quality of life, and somatic late effects. Special attention is given to survivors from diverse socio-demographic backgrounds, including those with a migration history, in order to explore the unique challenges they face.

**Discussion:**

The results of the study will contribute to the development of needs-based care recommendations for cancer survivors, particularly those in potentially vulnerable groups. The findings will inform the design of more inclusive care strategies and interventions, leading to better long-term health outcomes for cancer survivors in Germany.

**Trial registration:**

German Clinical Trials Register: DRKS00032146, registered on 03/12/2024.

## Background

The number of cancer survivors in Germany has been steadily increasing, due to advancements in treatment options and early detection [1]. In 2017 [2], there were 4.65 million cancer survivors, with approximately two-thirds being long-term survivors who have remained cancer-free for the last five years [3]. This increasing number of survivors spans all age groups, with improved therapies contributing significantly to this trend [4]. However, the majority of cancer patients treated in the 1970s, 1980s, and 1990s have not been adequately informed about the risks of late effects or the need for subsequent follow-up care. Most of the current knowledge about therapy-associated late effects has emerged in the past 20 years [5].

In 2019, approximately 117,518 new cancer diagnoses were recorded in North Rhine-Westphalia (NRW), Germany, excluding ICD-10 code C44 (other neoplasms of the skin). For the adult cancer patient population, a relative survival probability of over 60% is expected, aligning with the national average reported by the Robert Koch Institute [6]. However, the anti cancer treatment itself, along with intensive multimodal therapies, leads to late effects that can impact survivors’ health and quality of life, contributing to increased morbidity, mortality, and psychological distress [7]. These late effects are often associated with a latency period and can include life-long impairments, such as those resulting from major mutilating surgeries, stomas, prosthetics, blindness, and cognitive disorders [8, 9].

Furthermore, many long-term cancer survivors were not adequately informed about the potential risks of therapy-associated late effects and behavioral changes, such as a reduction in daily physical activity during their treatment, primarily due to the limited knowledge available at that time. Beyond the somatic symptoms, psychological, social, and economic factors frequently interfere with the daily functioning and well-being of cancer survivors [10–13].

There is limited evidence on how cancer survivors with a migration background access and experience post-treatment care, and whether existing support structures adequately address their needs. Recent studies [14–17] highlight persistent disparities in care among ethnic minority populations, which correlate with poorer long-term outcomes. Although informational and supportive resources are available, their actual use and accessibility within these groups remain insufficiently explored.

To address these gaps, the OPTILATER project (Optimal Long-Term Survival After Cancer) has been developed. This large-scale project aims to systematically evaluate the needs of cancer survivors, focusing on the late effects of cancer treatment and the follow-up care required for optimal long-term survival.

This project is being conducted collaboratively at multiple sites across North Rhine-Westphalia (NRW), Germany’s most populous federal state. NRW is characterized by a diverse population, including individuals with migration backgrounds, making it a suitable region for investigating potential disparities in survivorship care. Additionally, NRW closely mirrors the national average in terms of the proportion of residents with a migration background, making it broadly representative of Germany as a whole [18].

The OPTILATER project involves a consortium of partners, including the LVR-University Hospital Essen, the Cancer Registry of North Rhine-Westphalia (LKR NRW), the West German Cancer Center (WTZ) including its Patient Advisory Board, the Institute of Medical Informatics, Biometry and Epidemiology (IMIBE) at the University Hospital Essen, and the University Hospital Cologne.

**Fig. 1:**
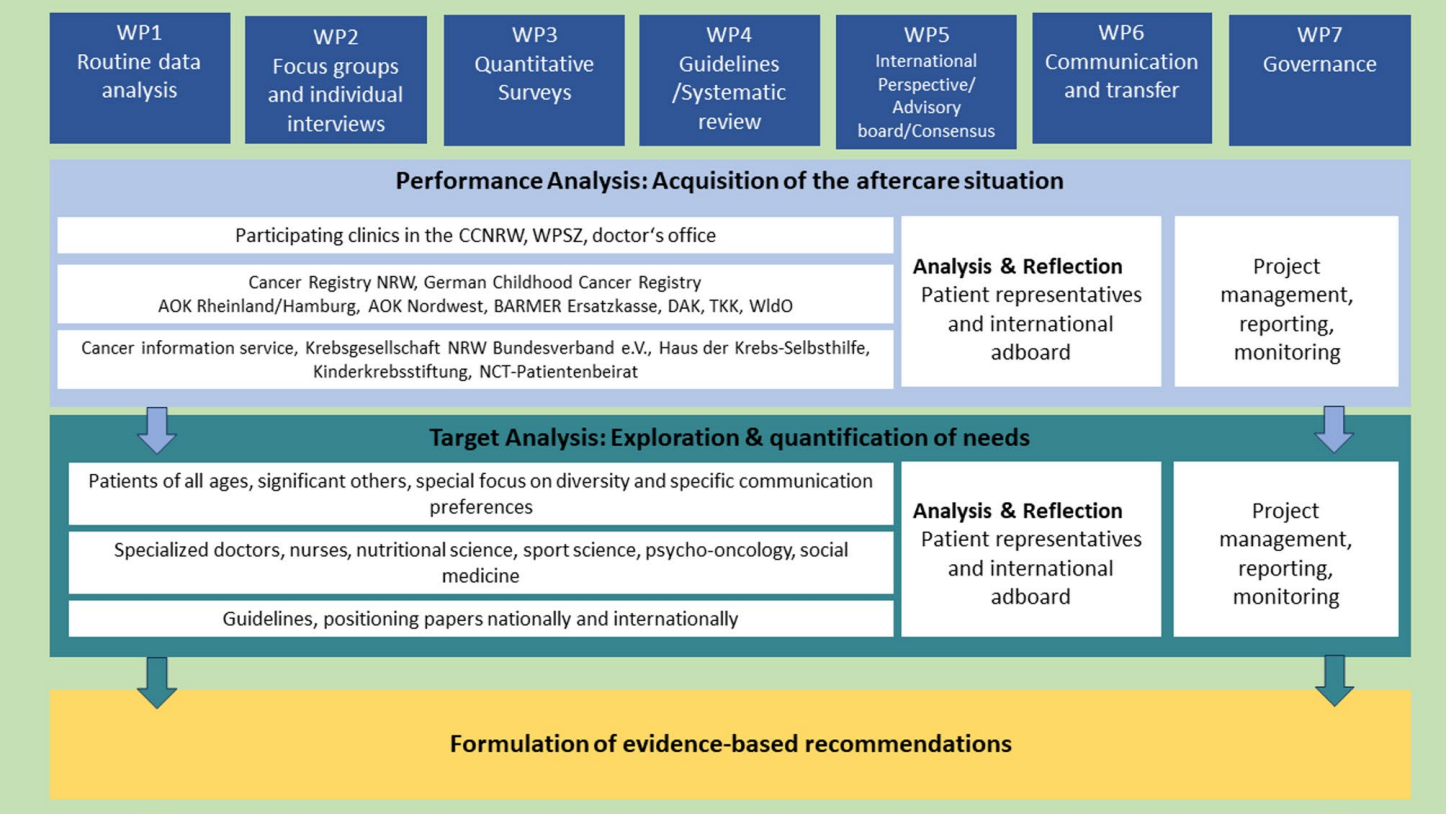
Work Packages (WP) within the OPTILATER Project

This study protocol focuses on Work Package 3 (WP3), which assesses the follow-up needs of adult cancer survivors within a larger framework comprising multiple other WPs (Fig. 1).

WP3 is currently collecting data on socioeconomic status, health-related quality of life, cognition, information status, psychological well-being (including depressive symptoms and anxiety), sleep, nutrition, physical activity, and somatic late effects. The study aims to assess unmet needs, including, among others, those related to digital health interventions, and to evaluate the health-related quality of life of survivors using established Patient-Reported Outcome Measures (PROMs).

Participant recruitment was carried out by the Cancer Registry of NRW (LKR NRW), ensuring comprehensive representation of the diverse survivor population in NRW, including individuals with migration backgrounds. All included participants have a confirmed history of cancer diagnosis.

By examining these areas, OPTILATER aims to provide evidence-based recommendations for improving survivorship care in Germany, especially for those from minority and underrepresented groups, and to develop targeted interventions for cancer survivors to enhance their long-term quality of life.

## Methods/design

### Study design

This study protocol is part of the OPTILATER project and uses a cross-sectional, survey-based design to assess the healthcare situation of long-term cancer survivors in Germany. WP3 specifically employs a quantitative survey methodology, integrating closely with other work packages to provide a holistic understanding of survivors’ care needs.

- Participants in WP3 will also be invited to express their interest in participating in qualitative interviews conducted as part of Work Package 2 (WP2), in order to facilitate a deeper exploration and contextualization of the quantitative findings.
- Initial qualitative insights from WP2 will inform secondary quantitative analyses in WP3, enriching the interpretation of survivors’ experiences and needs.
- A systematic literature review conducted in Work Package 4 (WP4) on long-term cancer care guidelines will underpin the conceptual framework of WP3, ensuring alignment with established best practices and current guidelines.

The interconnected design of this study supports a structured, multidimensional, and evidence-based approach aimed at enhancing long-term care for cancer survivors.

This exploratory study (WP3) employs a structured quantitative survey to systematically assess survivors’ experiences, unmet needs, and barriers to healthcare access. It does not include any medical or therapeutic interventions. The focus is on assessing the care situation and the experiences of long-term cancer survivors through structured questionnaires. No treatments, behavioral interventions, or clinical procedures are implemented as part of the study.

### Participants

The study aims to recruit up to 3,300 long-term cancer survivors, defined as individuals who have remained free from active tumor recurrence for at least five years following their initial diagnosis. The eligible tumor entities include colorectal cancer, lung (bronchial) cancer, prostate cancer, renal cell-cancer, female breast cancer, Hodgkin and non-Hodgkin lymphomas, leukemias, tumors of the central nervous system, bone and soft tissue sarcomas.

Participants were identified via the Cancer Registry of North Rhine-Westphalia (LKR NRW) database and invited to complete a structured quantitative survey questionnaire.

#### Inclusion criteria

Adults (≥ 18 years and < 75 years old) diagnosed with cancer between 2008 and 2018.

- Survival at least five years post-diagnosis.
- Resident of North Rhine-Westphalia, Germany at the time of diagnosis.
- Willing and able to provide informed consent.

#### Exclusion criteria

- Severe cognitive impairment that precludes meaningful participation.
- Insufficient proficiency in German (as the survey is administered in German).
- Residency outside of North Rhine-Westphalia, Germany at the time of invitation.

### Materials

The exploratory study used a range of validated instruments to assess various aspects of health and well-being among long-term cancer survivors:

EQ-5D (EuroQol Group, 1990) [19, 20]: Measures quality of life across mobility, self-care, usual activities, pain/discomfort, and anxiety/depression. Example item: “I have no problems walking about.“.

Patient-Reported Outcomes Measurement Information System (PROMIS) developed by the PROMIS Health Organization, as part of the National Institutes of Health (NIH), [20]: Includes scales for Depression [20, 21], Anxiety [22–24], Cognitive Functions [25], and Informational Support [26]. Example items: “I felt depressed,” “I felt anxious,” “I have trouble remembering things,” and “I have someone who provides helpful information.“.

Pittsburgh Sleep Quality Index (PSQI), [27, 28]: Evaluates sleep quality and disturbances over the previous month. Example item: “How often have you had trouble sleeping because you cannot get to sleep within 30 minutes?“.

General Dietary Behaviour Inventory (GDBI), [29]: Assesses dietary habits and eating behaviors using Likert-scale items. Example item: “I regularly eat fresh vegetables.“.

Global Physical Activity Questionnaire (G-PAQ), [30, 31]: Assesses physical activity levels and intensity. Example item: “Does your work involve vigorous-intensity activity that causes large increases in breathing or heart rate like [carrying or lifting heavy loads, digging or construction work] for at least 10 minutes continuously?“.

Euthyroid Socioeconomic Status Questionnaire, [32]: Measures socioeconomic indicators such as education, income, and occupation. Example item: “What is your highest completed educational level?“.

Migration Background Questionnaire (adapted from Youth Shell Study, [33, 34]): Evaluates migration status using standardized questions. Example item: “Were you or any of your parents born outside Germany?“.

Somatic Late Effects Questionnaire (developed collaboratively with the Children’s Cancer Registry and University Hospital Bonn): Identifies physical late effects related to cancer treatments. Example item: “Has a doctor ever diagnosed you with any of the following conditions or findings?”

Surveys were administered digitally via PROMspace (https://promspace.org) or through paper-based questionnaires with prepaid return envelopes. Sociodemographic data (age, sex, cancer type, and time since diagnosis) were provided by the LKR NRW in pseudonymized form and will be matched with survey data during quantitative analysis. Data collection involved self-reported questionnaires maintained in pseudonymized form to ensure confidentiality (Table 1).

**Table 1:** Questionnaires used in WP3 of the OPTILATER project

Measure	Domain	Sub-Domain
EQ-5D	Health-related Quality of Life	1. Mobility 2. Self-care 3. Usual activities 4. Pain/Discomfort 5. Anxiety/Depression
PROMIS Measures	Mental Health, Cogniton, Informational Support	1. Depression 2. Anxiety 3. Informational Support 4. Cognition
PSQI	Sleep Quality	1. Subjective sleep quality 2. Sleep latency 3. Sleep duration 4. Habitual sleep efficiency 5. Sleep disturbances 6. Use of sleeping medications 7. Daytime dysfunction
GDBI	General Dietary Behavior	
G-PAQ	Physical Activity	1. Physcial activity at Work 2. Travel to and from Places 3. Leisure-time physical activity
Sociodemographic data	-	
Based on questions from the Shell Youth Study	Migration	-
EUthyroid	Socioeconomic Status	-
Developed in Cooperation with the German Childhood Cancer Registry Mainz and the University Hospital Bonn (UKB)	Somatic Late Effects	-

### Procedure

Recruitment was facilitated via the Cancer Registry of North Rhine-Westphalia (LKR NRW), based on a confirmed cancer diagnosis, ensuring a representative sample of survivors. Potential participants were identified via the LKR NRW, which employs a structured approach to ensure the proportional representation of each tumor entity. Rare entities (e.g., bone and soft tissue sarcomas) were prioritized and sampled first. Subsequently, a random selection was conducted among the more prevalent target entities to achieve adequate representation across all groups. Participants received an initial contact to assess their willingness to participate. Those who agreed, signed an informed consent form and were officially enrolled in the study.

Participants’ socio-demographic and clinical data were sent in pseudonymized form from the LKR NRW to the LVR-University Hospital Essen. Separately, contact information was sent from the LKR NRW to the Institute for Medical Informatics, Biometry, and Epidemiology (IMIBE Essen). IMIBE Essen then distributed the questionnaires to participants. Participants were able to choose between completing digital questionnaires—accessible online via the PROMspace data platform using a QR code and a personalized link—or paper-based questionnaires, which were provided with prepaid return envelopes and did not include any personal contact information.

Participants returned the completed paper-based questionnaires directly to LVR-University Hospital Essen, where responses are maintained in a pseudonymized format throughout data processing. Alternatively, participants completed questionnaires online via PROMspace. Researchers will perform the data analysis using only pseudonymized participant ID numbers, ensuring that identifiable personal data are never accessible (Table 2).

**Table 2:** Study timeline and overview of Enrolment, Assessments, and data collection

Study period	Enrolment	Allocation	Post-allocation	Close-out
Timepoint	-t1	0	t1	t2
	*(Registry Identification)*	*(Consent / Study Inclusion)*	*(Survey Distribution & Completion)*	*(Data Analysis)*
Enrolment				
Eligibility screen	X (via Cancer Registry NRW)			
Contact & invitation	X (via Cancer Registry NRW)			
Informed consent		X (via Cancer Registry NRW)		
Allocation		Not applicable (observational design)		
Interventions				
*Not applicable (observational study)*				
Assessments				
Sociodemographic data			X (via IMIBE Essen)	
EQ-5D (Quality of Life)			X (via IMIBE Essen)	
PROMIS Measures			X (via IMIBE Essen)	
PSQI (Sleep Quality)			X (via IMIBE Essen)	
GDBI (Dietary Behavior)			X (via IMIBE Essen)	
G-PAQ (Physical Activity)			X (via IMIBE Essen)	
Socioeconomic Status			X (via IMIBE Essen)	
Migration Background			X (via IMIBE Essen)	
Somatic late effects			X (via IMIBE Essen)	
Data analysis				X (via LVR-University Hospital Essen)

## Discussion

The OPTILATER project aims to systematically address the needs of long-term cancer survivors in Germany by conducting a large-scale, structured, quantitative survey targeting various domains of survivorship. While advancements in cancer treatments have contributed to increased survival rates, many survivors continue to experience persistent physical, psychological, and social challenges that negatively impact their quality of life and overall well-being. The lack of structured, needs-oriented, and culturally sensitive follow-up care for these survivors underscores the necessity of this study.

The cross-sectional design of this study, with a target sample size of up to 3,300 long-term cancer survivors recruited by the Cancer Registry NRW, provides a robust framework for generating representative and generalizable findings. The recruitment strategy emphasizes inclusivity by prioritizing rare tumor entities while maintaining a balanced representation of more common diagnoses. This approach ensures that the perspectives of traditionally underserved and underrepresented patient groups are adequately captured. Furthermore, the geographic focus on North Rhine-Westphalia (NRW), Germany’s most populous federal state with a highly diverse population, enhances the generalizability of findings and allows for comprehensive assessments of disparities in survivorship care.

The utilization of validated instruments such as EQ-5D, PROMIS measures, PSQI, GDBI, and G-PAQ ensures that the data collected are reliable, comparable, and suitable for assessing a wide range of survivorship domains. By examining health-related quality of life, mental health, physical activity, nutrition, sleep quality, cognition, somatic late effects, and healthcare utilization, this study aims to provide a holistic understanding of survivors’ experiences. Additionally, by assessing digital health preferences and barriers, the study addresses the growing importance of telemedicine and other digital interventions in enhancing accessibility and efficiency of care.

A notable strength of the study lies in its comprehensive recruitment and data collection strategy, which incorporates both digital and paper-based survey options. This approach ensures accessibility for participants regardless of their technological proficiency or internet availability. The PROMspace platform provides a secure and convenient digital option, while the inclusion of paper-based surveys with prepaid return envelopes ensures that participants with limited digital literacy are not excluded.

This study has potential limitations. The cross-sectional design precludes the examination of changes in survivors’ needs and health status over time. Additionally, as participation is voluntary, there is a risk of nonresponse, particularly if certain groups, such as those with lower socioeconomic status or poorer health, are less likely to participate. To address this, a non-responder analysis will be conducted to identify systematic differences between respondents and non-respondents. Furthermore, the reliance on self-reported data may introduce recall bias or inaccuracies.

Sensitivity analyses and stratification by demographic and clinical characteristics will be performed to better understand the heterogeneity of survivor experiences.

The findings from AP3 will provide valuable insights into the current state of survivorship care in Germany, with a particular emphasis on vulnerable and underserved populations, such as individuals with migration backgrounds or those affected by rare tumor entities. By systematically identifying unmet needs, barriers, and gaps in care, the study aims to inform the development of targeted, evidence-based recommendations to enhance the accessibility, quality, and coordination of care for long-term cancer survivors.

Furthermore, the findings will complement qualitative insights gained from other work packages within the OPTILATER project, enhancing the overall understanding of survivorship experiences and informing the development of culturally sensitive and patient-centered interventions. Ultimately, this study aims to contribute to the improvement of survivorship care in Germany by providing a solid evidence base for future guidelines and policies.

Building on these insights, the OPTILATER project will translate the quantitative findings into actionable, evidence-based recommendations by engaging stakeholders across the healthcare system. This process aims to develop tailored care strategies that address identified needs and barriers, ultimately supporting the implementation of improved survivorship care policies and practices in Germany.

## Data Availability

No datasets were generated or analysed during the current study.

## Abbreviations

AP: Arbeitspaket (German for "Work Package") – e.g., AP3 = Work Package 3
DRKS: Deutsches Register Klinischer Studien (German Clinical Trials Register)
EQ-5D: EuroQol 5-Dimension (quality of life measure)
GDBI: General Dietary Behaviour Inventory
G-PAQ: Global Physical Activity Questionnaire
IMIBE: Institute for Medical Informatics, Biometry, and Epidemiology (Essen)
LKR NRW: Landeskrebsregister Nordrhein-Westfalen (Cancer Registry of North Rhine-Westphalia)
LVR: Landschaftsverband Rheinland (regional public health organization in NRW)
NIH: National Institutes of Health
NRW: North Rhine-Westphalia (federal state in Germany)
OPTILATER: Optimal Long-Term Survival After Cancer (project name)
PROMs: Patient-Reported Outcome Measures
PROMIS: Patient-Reported Outcomes Measurement Information System
PROMspace: A digital platform for survey administration (https://promspace.org)
PSQI: Pittsburgh Sleep Quality Index
WP: Work Package
